# Supplementary material for: Jolkinolide B inhibits the progression of hepatocellular carcinoma by regulating Musashi-2 protein
Source: PLoS One. 2024 Apr 17;19(4):e0299920. doi: 10.1371/journal.pone.0299920 (PMC11023458; doi:10.1371/journal.pone.0299920)

Original immunoblots for Figure 2A, 2B, 2E

A

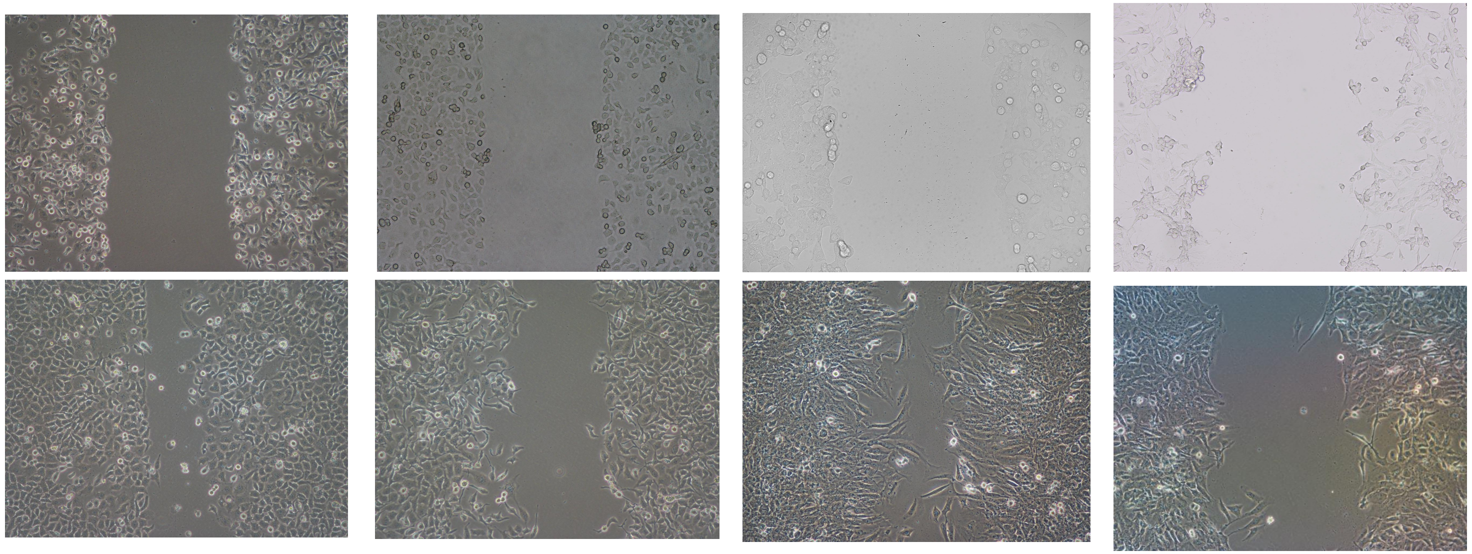

B

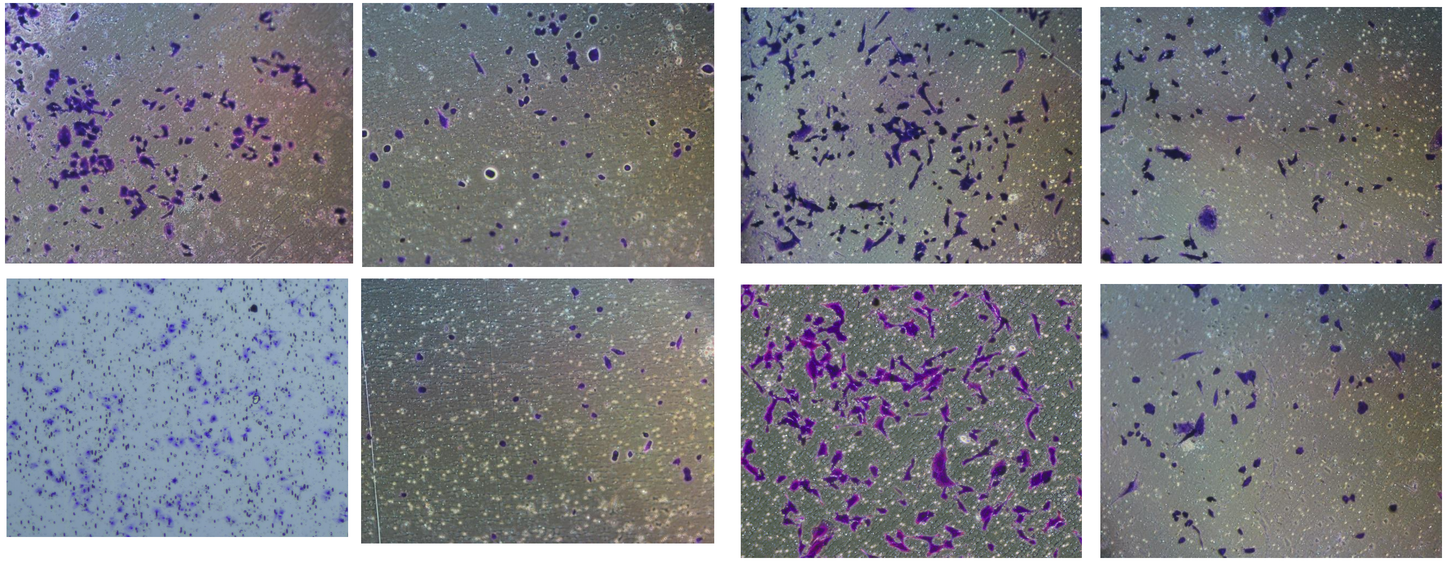

E

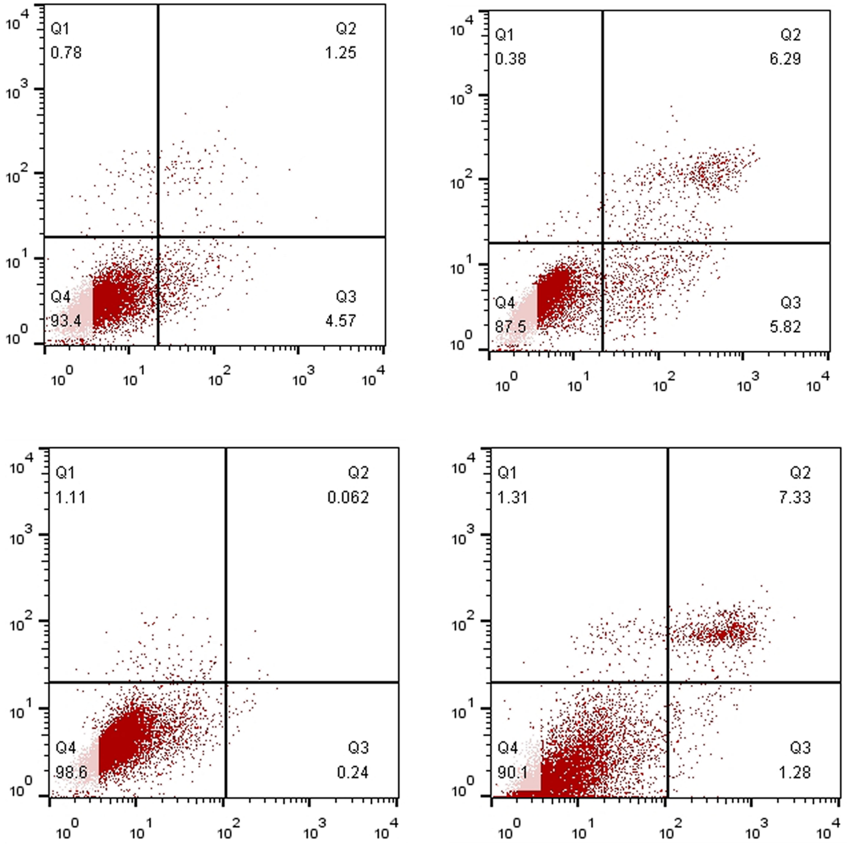

Original immunoblots for Figure 2C, 2D

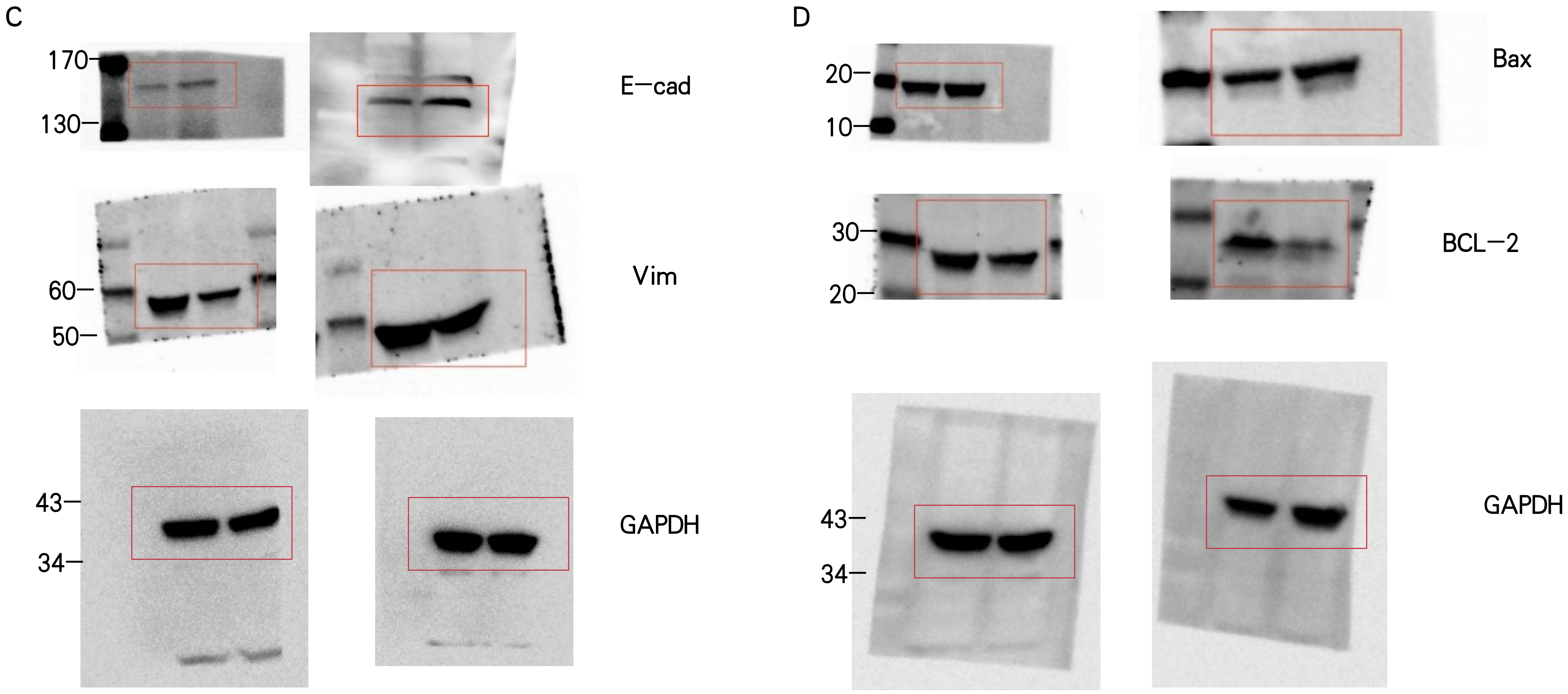

Original immunoblots for Figure 3

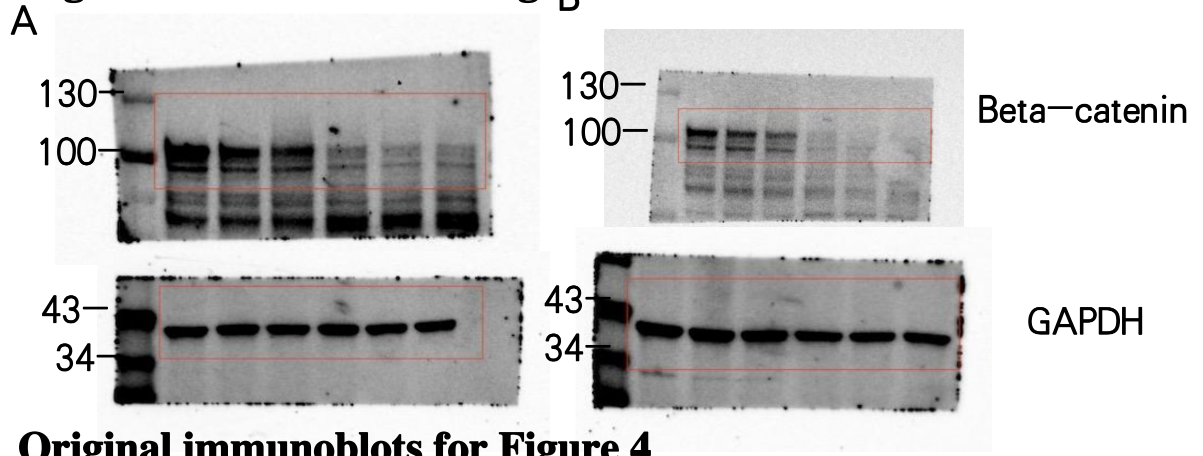

Original immunoblots for Figure 4

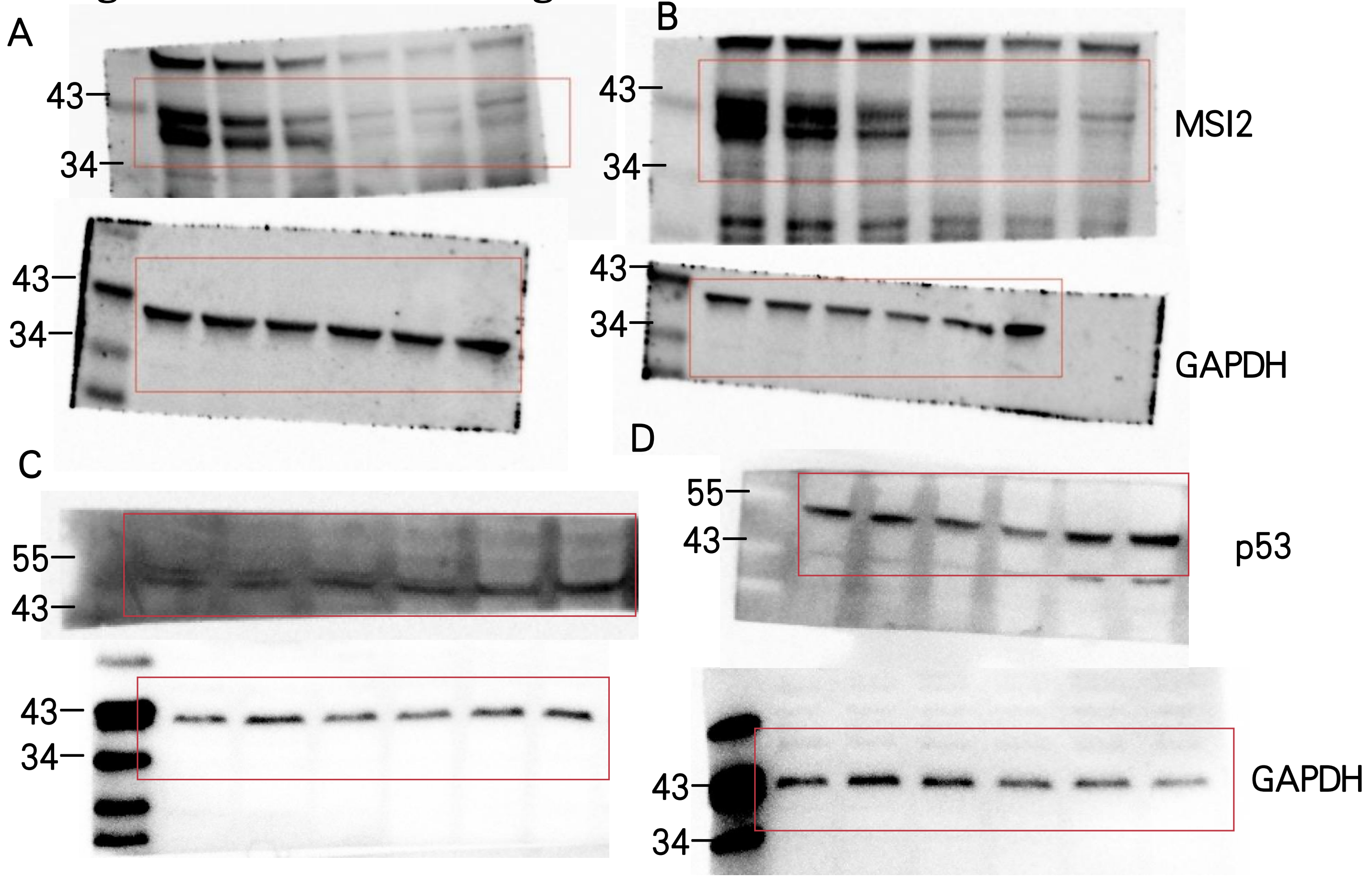

Original immunoblots for Figure 5

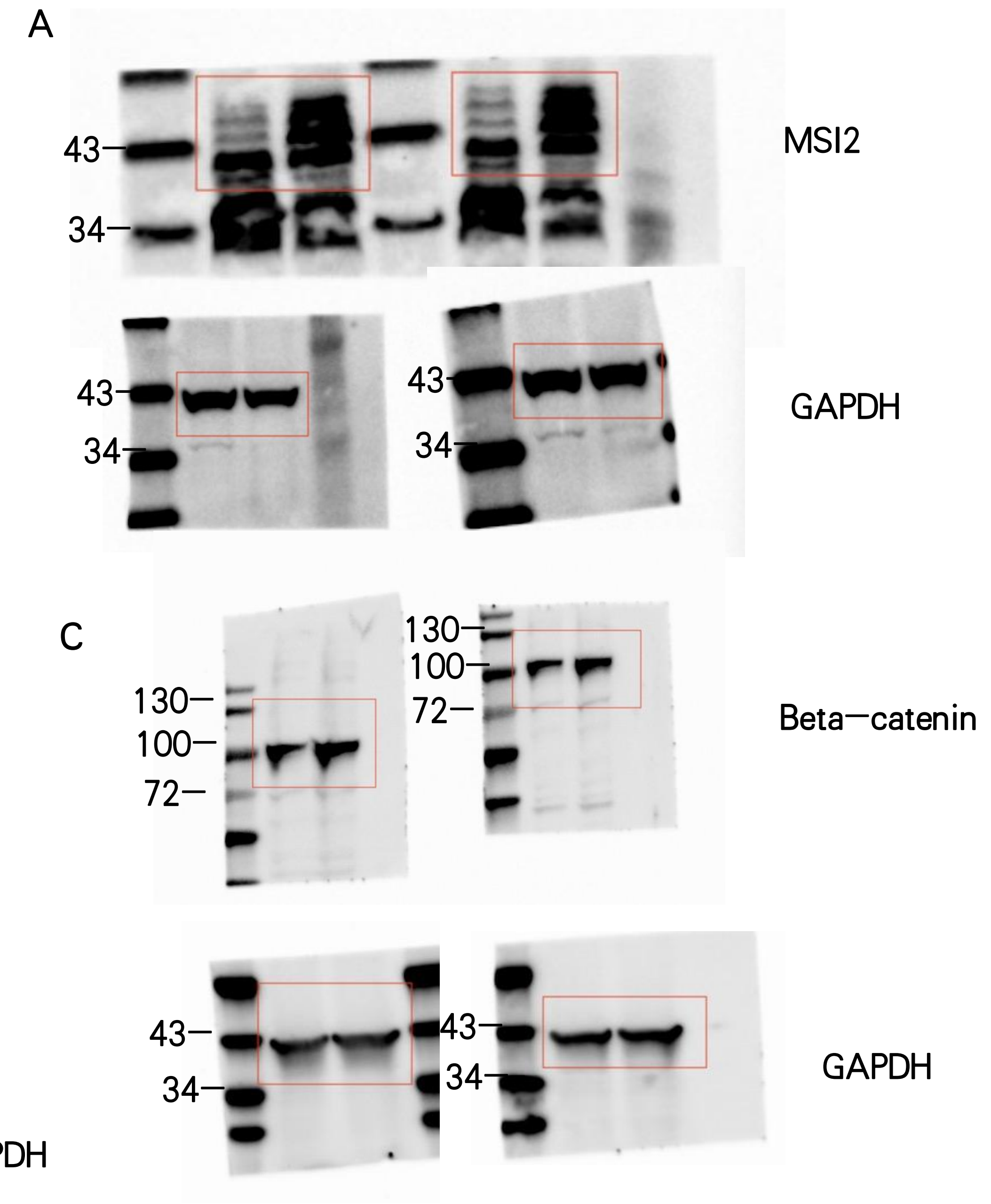

Original immunoblots for Figure 6

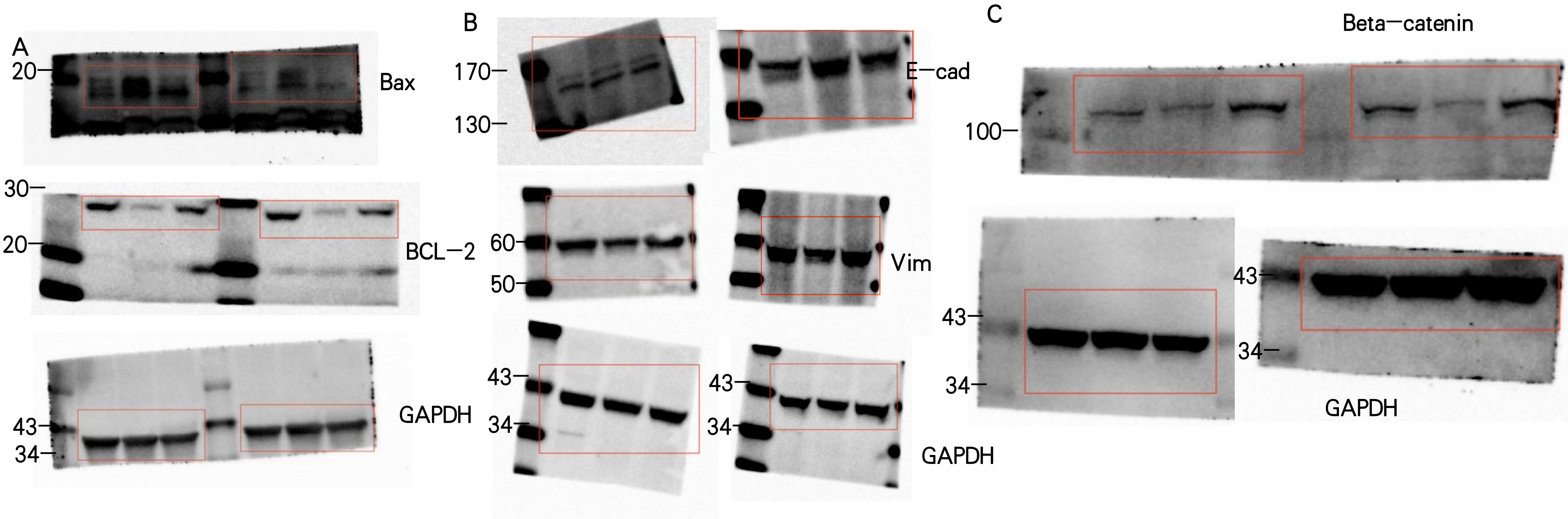

Supplement: S1 File — Slide 1: Original images for Fig 2A, 2B, 2E. Slide 2: Original immunoblots for Fig 2C, 2D. Slide 3: Original immunoblots for Fig 3. Slide 4: Original immunoblots for Fig 4. Slide 5: Original immunoblots for Fig 5A, 5C. Slide 6: Original immunoblots for Fig 6A, 6B. Slide 7: Original immunoblots for Fig 6C. Sheet 1: Original CCK8 of cell viability for Fig 1A-1C. Original RT-qPCR for Figs 3C, 3D, 5B. (ZIP) [file pone.0299920.s001.zip › raw image.pdf]
