# Supplementary material for: Jolkinolide B inhibits the progression of hepatocellular carcinoma by regulating Musashi-2 protein
Source: PLoS One. 2024 Apr 17;19(4):e0299920. doi: 10.1371/journal.pone.0299920 (PMC11023458; doi:10.1371/journal.pone.0299920)
Supplement: Attachment — Submitted filename: PONE-D-23-36751_comments.docx [file pone.0299920.s002.docx]

Based on the information presented in the study, the following concerns are proposed as prospective enhancements:

Major revision.

The manuscript represents the role of Jolkinolide B in the progression of hepatocellular carcinoma through the regulation of Musashi-2 protein; however, the authors did not investigate the mechanism in detail. They may detect the change in the survival signaling pathways such as STAT3, p53, AP-1, ATF6, or PI3K/AKT pathways.

Minor revision.

1. The manuscript is standard English, though some errors should be rectified.
2. In the legends of Figures 3 and 4, gapdh should be GAPDH, and also in materials and method of RT-qPCR.
3. Project No. in the funding section needs to be included.
