# Supplementary material for: Jolkinolide B inhibits the progression of hepatocellular carcinoma by regulating Musashi-2 protein
Source: PLoS One. 2024 Apr 17;19(4):e0299920. doi: 10.1371/journal.pone.0299920 (PMC11023458; doi:10.1371/journal.pone.0299920)
Supplement: Attachment — Submitted filename: Response to Reviewer.docx [file pone.0299920.s003.docx]

**Dear Editor and Reviewer:**

Thank you for your letter and for the reviewer’s comments concerning our manuscript entitled “Jolkinolide B inhibits the progression of hepatocellular carcinoma by regulating Musashi-2 protein”. We very much appreciate all of the comments received in the review. Those comments are all valuable and very helpful for revising and improving our paper, as well as the important guiding significance to our researchers. We have studied these comments carefully and have made corrections accordingly. Our responses are given in a point-by-point manner below, and marked by blue. Changes to the manuscript are shown with track changes.

We hope the revised version is now suitable for publication and look forward to hearing from you in due course.

**Responds to the reviewer’s comments:**

**Reviewer #1:** Based on the information presented in the study, the following concerns are proposed as prospective enhancements:

**Major revision.**

The manuscript represents the role of Jolkinolide B in the progression of hepatocellular carcinoma through the regulation of Musashi-2 protein; however, the authors did not investigate the mechanism in detail. They may detect changes in the survival signaling pathways such as STAT3, p53, AP-1, ATF6, or PI3K/AKT pathways.

**Reply:**

We thank the reviewer’s question and following the reviewer’s suggestion, as p53 protein having an important role in inhibiting cancer progression, we have now detected the p53 protein expression in Jolkinolide B-induced HCC cell lines in *this manuscript.*

**Minor revision.**

The manuscript is standard English, though some errors should be rectified.

**Reply:**

We thank the reviewer’s question. We have now reviewed and revised our manuscript with the help of native English-speakers.

In the legends of Figures 3 and 4, gapdh should be GAPDH, and also in materials and method of RT-qPCR.

**Reply:**

We thank the reviewer’s question. We have now revised the writings in the manuscript.

3. Project No. in the funding section needs to be included.

**Reply:**

We thank the reviewer’s question. The project number in the funding section has been included in the revised manuscript.

**Reviewer #2:** The manuscript entitled “Jolkinolide B inhibits the progression of hepatocellular carcinoma through regulating musashi-2 protein” provides new insights that, Jolkinolide B as a potential candidate against hepatocellular carcinoma. It needs major revision to make the suitable for publication.

IC_50_ value of Jolkinolide B should be calculated.

Reply:

We thank the reviewer’s question and the IC50 values of Jolkinolide B in HCC cell lines have been calculated.

2.Authors should confirm the apoptosis induced by Jolkinolide B, Annexin V or Tunnel assay in both lines.

**Reply:**

We thank the reviewer’s question. We have detected the apoptosis of HCC cell lines treated by DMSO or Jolkinolide B administration by flow cytometry.

3. In figure 2 legend, authors should mention concentration of JB and time of treatment of better understanding.

**Reply:**

We thank the reviewer’s question and we are sorry for having not described the experiments more clearly. We have now provided the description of Jolkinolide B concentration and time of treatment in the revised manuscript.

4. Similarly in Figure 3 and 4 legends, mention the treatment time.

**Reply:**

We thank the reviewer’s question and we are sorry for having not described the experiments more clearly. We have now provided more precise description of Jolkinolide B’s treatment time in the revised manuscript.

5. Authors also should include the effect of JB on normal hepto cell lines.

**Reply:**

We thank the reviewer’s question and we now have detected the effect of Jolkinolide B on normal hepto cell line.

6. All Western blots in manuscript should be quantified.

**Reply:**

We thank the reviewer’s question and following the reviewer’s suggestion, we have quantified the normalized intensity band ratios of all western blots in revised manuscript.
